# Effects of ginsenoside Rg1-loaded alginate-chitosan microspheres on human bone marrow stromal cells

**DOI:** 10.1042/BSR20160566

**Published:** 2017-06-27

**Authors:** Yu-Hai Guo, Shuai Zhao, Yan-Xin Du, Qing-Jia Xing, Bo-Lai Chen, Chu-Qin Yu

**Affiliations:** 1 Department of Orthopedics, Guangdong Provincial Hospital of Traditional Chinese Medicine, Guangzhou 510120, China; 2 College of Pharmacy, Guangdong Pharmaceutical University, Guangzhou 510006, China

**Keywords:** alginate-chitosan microspheres, differentiation, Ginsenoside Rg1, Human bone marrow mesenchymal stem cells, proliferation

## Abstract

The ginsenoside Rg1 is the most abundant compound in ginseng. Recent studies showed that Rg1 had neuroprotective effects on neuronal cells. The present study was to prepare Rg1-loaded alginate-chitosan microspheres and research the effects of microspheres on human bone marrow (BM) stromal cells (hBMSC). The alginate-chitosan microspheres were prepared by mechanical emulsification technique in combination with ion (Ca^2+^) and chitosan solidification. Subsequently, the microspheres were employed to load Rg1 ginseng extracts. The microspheres had a smooth surface and were spherical in shape. The average diameter of the microspheres was 3.95 µm. The loading efficiency was approximately 2.12%. The purity of isolated hBMSC was over 98.8%. Rg1-loaded microspheres could promote hBMSC proliferation and differentiation. Meanwhile, Rg1-loaded microspheres could also suppress hBMSC apoptosis induced by hypoxia-reoxygenation. In conclusion, these loaded microspheres may be used in the research of neuroprotective effects of Rg1.

## Introduction

Ginseng is one of the most common medicinal plants used in China for thousands of years to produce various pharmacological and biological effects. The most important components contributing to its multiple medicinal properties are the ginsenosides [1]. The previous studies reported that ginsenosides had neuroprotective effects on central nervous system disorders and neuronal diseases [2,3]. Lee et al. [4] found that cognitive behavior in patients with Alzheimer’s disease was improved by ginseng powder. Ginseng extract prevented the development of locomotion deficits in patients with Parkinson’s disease [5]. Up to now, over 150 kinds of ginsenosides have been isolated from roots, stems, flowers, leaves and even fruits of the ginseng species [6,7]. Ginsenoside Rg1, a steroid-like structure with the sugar moieties modification, is one of the most active components of Panaxatriol saponins.

Alginate is a natural polymer extracted from the brown algae. It is a biodegradable and biocompatible polymer, easily functionalized. Recently, the alginate has been used as a carrier material for protein, peptide and small molecule drugs due to its excellent biocompatibility, dissolvability, biodegradability and mild gelation conditions. This polymer has shown interesting characteristics in many fields, from pharmaceutical and drug delivering and releasing applications to tissue engineering [8,9]. Alginate has been used as a carrier material in oral drug delivery system design [10,11], and it has been formulated as microspheres, gel beads, microcapsules, film, hydrogel, tablets and nanoparticles [12].

Bone marrow (BM) stromal cells (BMSC) were initially identified in the BM as multipotent, non-haematopoietic progenitor cells that can differentiate into osteoblasts, chondrocytes, adipocytes, tenocytes, visceral mesodermal cells and skeletal myocytes [13]. BMSC are the precursors of different mesenchymal tissues that play an important role in the construction of normal and pathological microenvironments [14]. BMSC can influence haematopoietic stem cell homing and differentiation through cell–cell interaction and by the production of chemoattractants and cytokines [15]. They can also be used to regenerate bone, myocardial, cartilage and hepatic tissues [16]. Lu et al. [17] found that ginsenoside Rg1 could significantly promote the proliferation of cultured porcine BMSC in a concentration-dependent manner. The recent study demonstrated that Rg1 improved myelosuppression through its action on the proliferation and migration of progenitor cells and haematopoietic stem from the spleen to the BM [18].

Therefore, we infer that Rg1-loaded alginate microspheres may have important effects on proliferation and differentiation of human BMSC (hBMSC). And our study aimed to find the potential role of Rg1-loaded alginate-chitosan microspheres, which would lay an important experimental foundation for their clinical application. In our study, first, the mechanical emulsification technique was employed to prepare alginate-chitosan microspheres for confirming the effects inferred. Then the particle size and surface morphology of microspheres were measured in detail. Moreover, then the ginseng extract Rg1 was loaded in the microspheres, and performed the further evaluations of Rg1-loaded alginate-chitosan microspheres in isolated hBMSC *in vitro*.

## Materials and methods

Ginsenoside Rg1 standard control (HPLC >98%) was purchased from the National Institute for the Control of Pharmaceutical and Biological Products (Beijing, China); ginsenoside extract Rg1 was prepared by Guangdong College of Pharmacy. Five total cases of rib were derived from the healthy and adult patients.

The present study was approved by the Institutional Review Board of Guangdong Provincial Hospital of Traditional Chinese Medicine (number: GDTCM1524), and all the participants gave written informed consents.

### Preparation of blank alginate-chitosan microspheres

The emulsification method was used for the preparation of microspheres followed by cross-linking with calcium chloride [19,20]. Various variables like sodium alginate and calcium concentration, cross-linking agent concentration, time and stirring rate required for cross-linking were considered in the optimization of the formulation. The liquid paraffin containing 3% (v/v) Span 80 was stirred using a mechanical stirrer (RCT B S25, IKA, Germany) at 900 rpm for 10 min, 2 ml of 3% sodium alginate in deionized water was added at 900 rpm and stirred for 10 min, and then added into 2 ml of 3% chitosan in deionized water stirring for 10 min, 3 ml of 3% calcium chloride dissolved in deionized water was added slowly to the above emulsion and stirred to assure efficient cross-linking. Supernatant was removed after centrifugation at 6000 rpm for 20 min. Then, the microspheres was resuspended and washed with abies oil, 95% absolute ethanol and deionized water respectively. This was repeated for three times and finally air-dried at room temperature. It was partially shipped in small bottle after drying, and then disinfection was done by irradiation with 3 kGy Co^60^.

### Ginsenoside Rg1 loading

The ginsenoside extract Rg1 (2 mg) was dissolved in 3% sodium alginate aqueous solution (2 ml). The liquid paraffin containing 3% (v/v) Span 80 was stirred at 900 rpm for 10 min, 2 ml of 3% sodium alginate dissolving ginsenoside extract Rg1 was added at 900 rpm and stirred for 10 min. The subsequent steps were the same as the preparation of blank alginate-chitosan microspheres.

### Analysis of the Rg1 in alginate-chitosan microspheres

Twenty milligrams blank and Rg1-loaded microspheres were completely dissolved in 6% sodium citrate solution, and filtered with the 0.45-µm pore filter (Millipore, MA, U.S.A.). The original concentration of ginsenoside Rg1 standard control solution (stock solution) was 1.037 mg/ml. Quantification of Rg1 was performed by using the HPLC system with fluorescence detection on an ACQUITY® UPLC HSS T3 column (2.1 × 50 mm, 1.8 μm). The mobile phase consisted of acetonitrile (mobile phase A) and 0.05% Na_2_HPO_4_ (PB, pH: 7, mobile phase B). Mobile phase A was elevated from 21 to 40% within 25 min with a flow rate of 0.3 ml/min. The detection wavelength was selected at 203 nm.

After these five different concentrations of Rg1 (the solutions of standard control) were separated and well prepared, the HPLC assay was processed. The sample volume is plotted as a horizontal co-ordinate, and the peak area is plotted as a vertical co-ordinate, and then the regression equation was calculated. HPLC assays analysed the Rg1-loaded microspheres. The peak area was substituted into the standard curve to obtain the Rg1 content.

### Isolation and culture of hBMSC

The isolation process was operated by following these published references [20–22]. BM was flushed from the adults’ ribs using the 23-gauge needle. BM mononuclear cells were separated by horizontal gradient density centrifugation (Guangzhou Youdi Biocompany, China) and cultured in Dulbecco’s modified Eagle’s medium (HyClone, U.S.A.) supplemented with 20% FBS (HyClone, U.S.A.), 100 U/ml penicillin and 100 U/ml streptomycin at 37°C and 5% CO_2_ atmosphere (Thermo, U.S.A.). Seventy-two hours later, nonadherent cells were removed and the adherent cells were split at 70–80% confluence (0.05% trypsin, Gibco, U.S.A.) and expanded in another flask. A homogeneous cell population was obtained after 2–3 weeks of continuous culturing.

### Flow cytometric analysis of hBMSC

Actually, hBMSC can not express both CD11a and CD14 proteins but can express CD29 protein. The identification of hBMSC was performed by flow cytometry using fluorescently labelled antibody. hBMSC were collected in PBS solution in pH 7.4. After washing with PBS, cells were resuspended in the permeabilization (0.1% saponin in PBS) solution. Subsequently, the cells were incubated with anti-CD11a-PE/Cy7 (Abcam, ab51220, U.S.A.), anti-CD14-PerCP (Abcam, ab91146, U.S.A.) and anti-CD29-FITC (Abcam, ab21845, U.S.A.) respectively. After washing with PBS, the cells were analysed on the FACSCalibur flow cytometer (BD, Accuri C6, U.S.A.).

### MTT assays

Cells were grown in DMEM supplemented with 20% FBS and 10 ng/ml (pre-experiment determined concentration) Rg1-loaded microspheres or blank microspheres respectively. The third-generation of hBMSC (1 × 10^3^) were seeded into 96-well plates and incubated at 37°C in 5% CO_2_ for 24 h. The MTT working solution was added into the wells, and the cells were incubated for 2–4 h. The medium was removed, and 150 μl of DMSO was added to dissolve the formazan crystals. Cell viability was assessed daily by absorbance at 490 nm using a microplate reader (model 680 Microplate Reader, Bio–Rad) for 24, 48 and 72 h. The proliferation assay was performed in three wells per time and repeated three times.

### Quantitative RT-PCR and flow cytometric analysis of differentiated hBMSC

The dried and sterilized Rg1-loaded microspheres and blank microspheres were soaked in DMEM medium for 3 days. The third passages of hBMSC were used into the induction of differentiation experiment. At first, the complete medium with 10 μg/l bFGF was employed to pre-induce for 24 h in hBMSC; then the serum-free medium containing Rg1-loaded or blank microspheres was added and induced for 3 h after three times washing with PBS. Then, the cells were co-incubated with anti-Nestin-FITC (Abcam, ab187846, U.S.A.) and anti-glial fibrillary acidic protein (GFAP)-Alexa Fluor® 647 (Abcam, ab194325, U.S.A.), and independently incubated with anti-NSE Alexa Fluor® 488 (Abcam, ab199775, U.S.A.) respectively. After washing with PBS, the cells were analysed on the FACSCalibur flow cytometer (BD, Accuri C6, U.S.A.).

Meanwhile, total RNA was extracted from hBMSC by using TRIzol reagent (Invitrogen). The cDNA synthesis kit was used to synthesize the cDNA according to the manufacturer’s instructions. Quantitative RT-PCR was performed to detect the expression level of mRNA. The primers used for quantitative RT-PCR are shown in Table 1. Quantitative PCR was accomplished to detect the expression levels of mRNA using the LightCycler 480 detection system (Roche Diagnostics, Indianapolis) and interaction dye SYBR Green. The quantitative RT-PCR results were analysed and expressed as relative mRNA levels of the CT value, which were then converted to fold change.

**Table 1:** Primers used in the present study

Genes	Names	Primers (5′–3′)
*Nestin*	Nestin-F	CTGCTACCCTTGAGACACCTG
	Nestin-R	GGGCTCTGATCTCTGCATCTAC
*NSE*	NSE-F	AGCCTCTACGGGCATCTATGA
	NSE-R	TTCTCAGTCCCATCCAACTCC
*GFAP*	GFAP-F	AGGTCCATGTGGAGCTTGAC
	GFAP-R	GCCATTGCCTCATACTGCGT
*GAPDH*	GAPDH-F	ACAACTTTGGTATCGTGGAAGG
	GAPDH-R	GCCATCACGCCACAGTTTC

Primers were designed using Primer Express version 2.0 software. Primer specificity was confirmed using Primer-BLAST web software (National Centre for Biotechnology Information).

### Hypoxia-reoxygenation treatment and Western blot analysis

hBMSC cultured in DMEM supplemented with 10% FBS and 10 ng/ml Rg1-loaded microspheres or blank microspheres were treated with hypoxia-reoxygenation. The cells were first incubated under hypoxia using a 95% N_2_ and 5% CO_2_ for 5 h at 37°C in a water-jacketed N_2_/CO_2_ incubator and then incubated in a standard incubator with 5% CO_2_ in normal air at 37°C for 20 h. Then, the cells were harvested and washed with ice-cold PBS three times, lysed with ice-cold RIPA lysis buffer (Beyotime, China) with 1 mmol/l PMSF. The protein concentrations were tested by BCA Protein Assay Kit (Thermo Fisher Scientific, U.S.A.). Twenty five micrograms of extracted protein were separated by SDS/PAGE (12% (w/v) gel) and transferred to PVDF membranes (Thermo Fisher Scientific, U.S.A.). The membrane was blocked with 5% nonfat dry milk in 0.1% Tween-20 (TBST) at room temperature for 1 h and immunoblotted with anti-Bax (Abcam, ab32503) and anti-Bcl-2 (Abcam, ab117115) at 4°C overnight, followed by incubation with the appropriate horseradish peroxidase–conjugated secondary antibodies for another 1 h at room temperature. After washing five times with PBS, the blots were treated with enhanced chemiluminescent substrate (Thermo Fisher Scientific, U.S.A.) and then exposed to the ChemiDoc MP system (Bio–Rad Laboratories, U.S.A.). Densitometry was performed using Image Lab Software and normalized to GAPDH control.

### Statistical methods

SPSS17.0 statistical software was used for statistical analysis. The significance was determined with the Student’s *t* test or one-way ANOVA. The measurement data were represented as mean ± S.D. (X ± s). *P*<0.05 indicated statistically significant differences.

## Results

### Surface morphology analysis of alginate-chitosan microspheres

**Figure 1:**
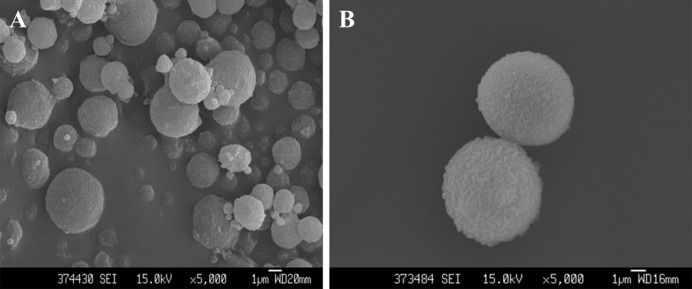
SEM images of two groups of microspheres. SEM images of blank alginate-chitosan microsperes(A) and Rg1-loaded microspheres(B)

The shape and surface characteristics were determined by SEM (Hitachi, SU3500). Surface morphology and particle sizes of both blank and Rg1-loaded microspheres were measured. We chose more than 100 particles in five different fields for measurement. In Figure 1A, the blank microspheres have smooth surface and appear spherical in shape. Moreover, the morphology of Rg1-loaded microspheres was similar to the blank microspheres (Figure 1B). The average diameter of the microspheres was 3.95 µm.

### The Rg1-loaded rate in alginate-chitosan microspheres

**Figure 2:**
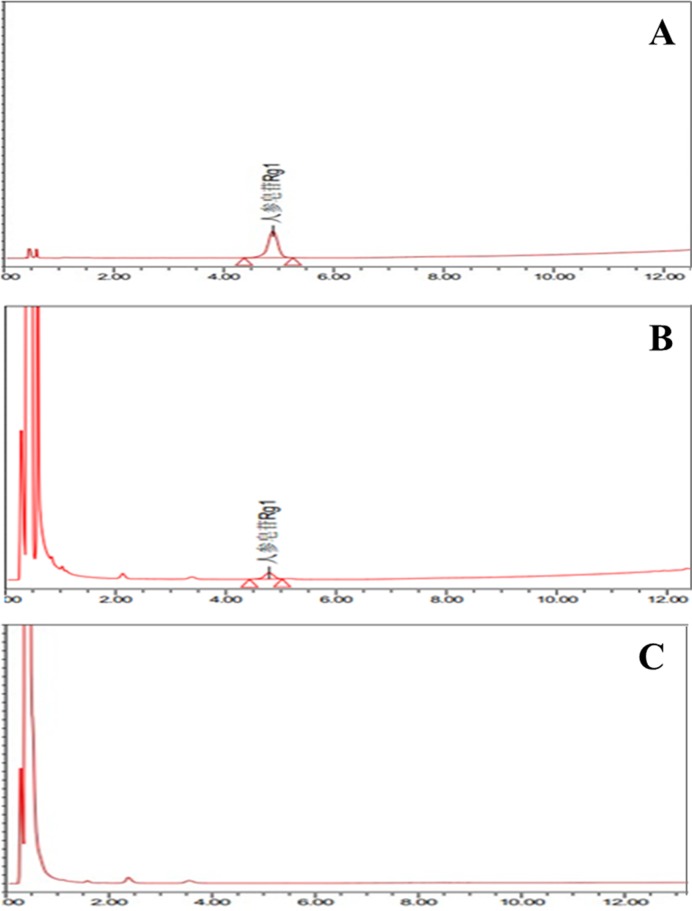
Chromatograms data of three groups. (**A**) The Rg1 control group. (**B**) Rg1-loaded microspheres group. (**C**) Blank microspheres group.

Chromatograms are shown in Figure 2. The retention time of Rg1 in the ginsenosides control group was consistent with the Rg1-loaded microspheres group. In addition, the chromatographic peak of blank microspheres was consistent with Rg1 control group and Rg1-loaded microspheres, which indicated that the blank microspheres themselves had no interference into HPLC assays. The above results indicated that Rg1 was successfully loaded in the blank microspheres.

The regression equation of Rg1 standard solution was Y =949132X + 4004, *R^2^*=0.9996, linear range was 0.08240–0.8240 μg/mg. Rg1 standard control had good correlation in its linear range. The peak area of Rg1-loaded microspheres was substituted into the above regression equation, then we obtained the Rg1-loaded rate in alginate-chitosan microspheres as 2.12% (w/w).

### Purity of isolated hBMSC

**Figure 3:**
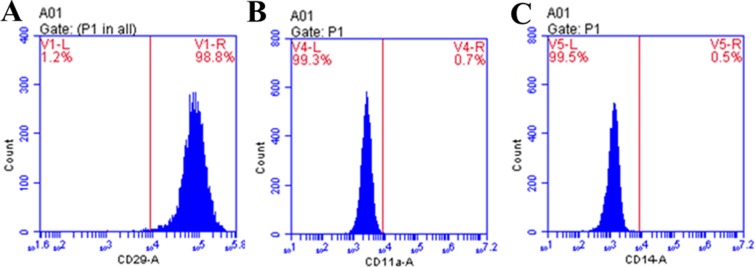
Flow cytometric analysis of the isolated hBMSC. (**A**) CD29: right panel exhibits the percentage of positive CD29 present. Left panel indicates the percentage of negatively presented CD29. (**B**) CD11a: right panel shows the percentage of positive presentation of CD11a present. Left panel demonstrates the percentage of negative CD11a present. (**C**) CD14: right panel exhibits the percentage of positive CD14 present. Left panel indicates the percentage of negatively presented CD14. Results are representative of three independent experiments.

As shown in Figure 3, 98.8% of hBMSC could express CD29, and only 0.7 and 0.5% of hBMSC could express CD11a and CD14 respectively. Compared with the blank microspheres group, the difference was significant (*P*<0.05). So the purity of hBMSC (98.8%) was high enough to experiment later.

### Rg1-loaded microspheres promote hBMSC proliferation

**Figure 4:**
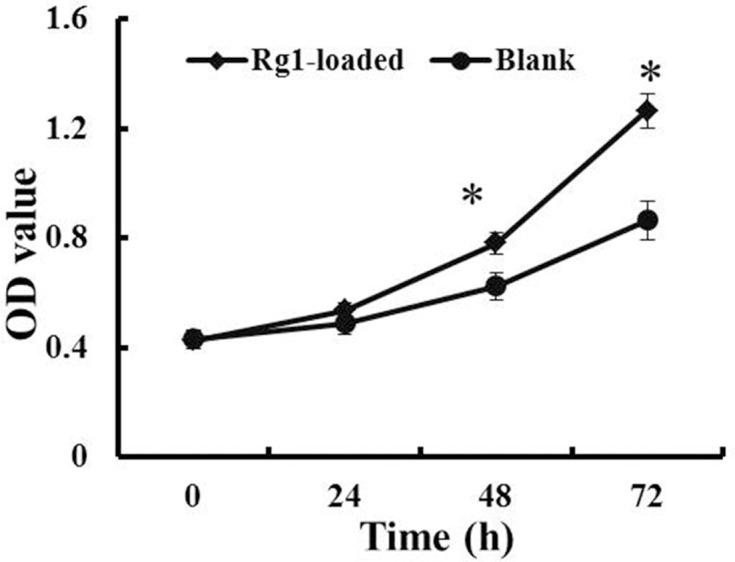
The proliferation capacity of hBMSC after Rg1-loaded microspheres treatment by MTT assay. Data are expressed as mean ± S.D., *n*=3. *P<0.05, compared with blank microspheres group.

The results are depicted in Figure 4. Compared with blank microspheres group, treatment with 10 ng/ml Rg1-loaded microspheres appeared to increase the proliferation capacity at 24, 48 and 72 h. At 48 and 72 h, the numbers of hBMSC in the Rg1-loaded microspheres group were significantly higher than that in blank microspheres group respectively (*P*<0.05). These results meant that Rg1-loaded microspheres could promote hBMSC proliferation.

### Rg1-loaded microspheres promote hBMSC differentiation

**Figure 5:**
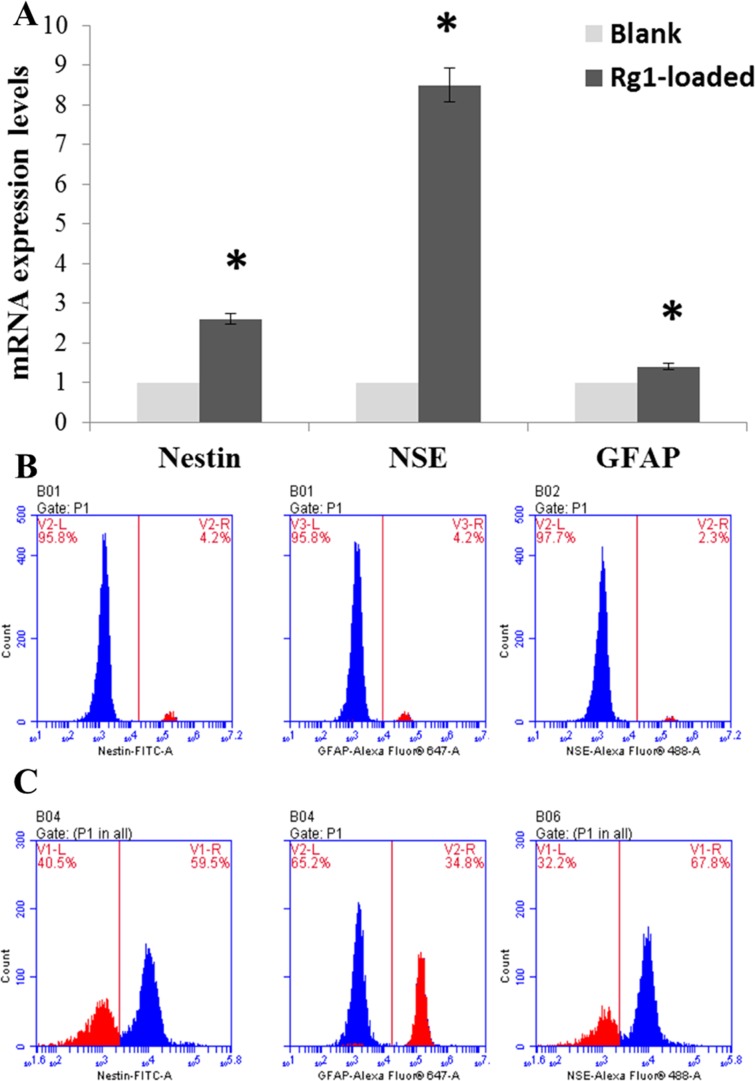
Relative mRNA expression levels of specific marker related genes in hBMSC and flow cytometric analysis of the differentiated hBMSC. (**A**) Quantitative real-time PCR detected mRNA expression of nestin, NSE and GFAP. GAPDH was used as control. * indicates significant intergroup difference when compared with the blank microspheres group (*P<0.05). (**B**) The percentages of differentiated hBMSC in blank microspheres group. (**C**) The percentages of differentiated hBMSC in Rg1-loaded microspheres group.

As we all know, GFAP is a specific marker protein of glial cells, NSE is an acidic protease that is unique to neurons and neuroendocrine cells, and Nestin is a characteristic marker of neural precursors. In comparison with the blank microspheres group, treatment with 10 ng/ml Rg1-loaded microspheres for 3 h obviously induced the mRNA expression of nestin, NSE and GFAP (Figure 5A, *P*<0.05). Moreover, compared with the blank microspheres group, the percent of nestin-, NSE- and GFAP-positive cells were 59.5, 67.8 and 34.8% respectively, the differences were significant (Figure 5B and 5C,* P*<0.05). This meant that Rg1-loaded microspheres could promote the differentiation of hBMSC into neural stem cells, neuronal cells and glial cells.

### Rg1-loaded microspheres suppress hBMSC apoptosis

**Figure 6:**
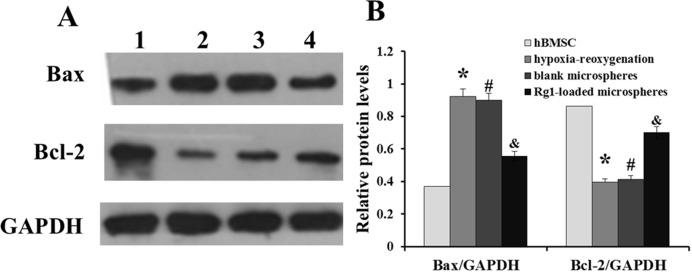
Western blots analysis of Bax and Bcl-2 expression. (**A**) Schematic image showing Western blotting. 1: hBMSC group, 2: hypoxia-reoxygenation treatment group, 3: blank microspheres and hypoxia-reoxygenation treatment group, 4: Rg1-loaded microspheres and hypoxia-reoxygenation treatment group. (**B**) Relative protein levels of Bax and Bcl-2. GAPDH was used as a loading control in each assay. The bar graph represents the densitometric analysis of three different experiments. *P<0.05, compared with hBMSC; ^#^*P*<0.05, compared with blank microspheres group. ^&^*P*<0.05, compared with hypoxia-reoxygenation treatment group.

As shown in Figure 6A, the pro-apoptotic protein Bax of hBMSC had a low expression level, and anti-apoptotic protein Bcl-2 had a high expression level. When hBMSC were treated with Rg1-loaded microspheres and hypoxia-reoxygenation for 48 h, Bax expression level increased and Bcl-2 expression level decreased, compared with only hypoxia-reoxygenation treatment group. However, when hBMSC were treated with blank microspheres and hypoxia-reoxygenation for 48 h, Bax and Bcl-2 expression had no obvious changes (Figure 6B). These results implied that Rg1-loaded microspheres could suppress hBMSC apoptosis caused by hypoxia-reoxygenation treatment.

## Discussion

In the present study, we found that Rg1 could promote hBMSC proliferation and differentiation, and suppress hBMSC apoptosis induced by hypoxia-reoxygenation. In clinical, major human brain and spinal cord injury remain having serious problems and moreover, it still has no effective treatments. Almost all of the central nervous system injuries are accompanied by neuronal apoptosis [23]. In ischaemic stroke, traumatic brain injury and traumatic spinal cord injury in rodent models, transplantation of hBMSC into the injured brain or spinal cord provides therapeutic benefits [24]. The potential mechanisms of action of transplanted adult hMSCs in CNS injury include differentiation to replace damaged neural cells (neurons, astrocytes, oligodendrocytes), neuroprotection (reduction in apoptosis), and creation of a favourable environment (proliferation of endogenous neural progenitors, proliferation of endogenous oligodendrocytes and so on) [25]. Therefore, with the promotion of cell proliferation, differentiation and inhibition of apoptosis of Rg1, it may play a positive role in transplantation of hMSCs. Rg1 has been extensively reported to exert neuroprotective and neurotrophic effects on the central nervous system both *in vivo* and *in vitro*. Lu et al. [26] found Rg1 enhanced BMSC proliferation through the activation of the oestrogen receptor mediated signalling pathway. Wang et al. [27] found that ginsenoside Rg1 promoted the proliferation and differentiation of human dental pulp cells. A variety of mechanisms underlying Rg1 have been identified, including the activation of nerve growth factor, antioxidant, anti-apoptotic, anti-inflammatory and immune stimulatory activities, the inhibition of excitotoxicity and excessive Ca^2+^ influx into neurons, maintenance of the cellular ATP levels, and the preservation of the structural integrity of neurons [28,29].

The small molecule drugs loaded into the microspheres have a unique advantage, which is a hot topic in drug delivery administration [10,12]. Alginate-based materials have become an important class of products in the pharmaceutical industry, because of their ability to create stimuli-responsive hydrogels, polymer degradation, relying on diffusion or using responsive polymer properties to trigger the release of the molecules. These microsphere diameters usually range from 1 to 10 μm [30]. The average diameter of microspheres was 3.95 µm with smooth surface, and Rg1 slowly released within 48 h, and the cumulative release rate reached 85.41% (which are not shown in the text). These experimental results indicated that the microspheres were available.

In order to simultaneously utilize Rg1 and microspheres, Rg1 were loaded with alginate-chitosan microspheres in the present study. When the isolated hBMSC were treated with these Rg1-loaded microspheres, the proliferation ability increased, and the hBMSC differentiated into neural stem cells, neuronal cells and glial cells. Compared with blank alginate-chitosan microspheres treatmeat group, the results implied that Rg1-loaded alginate-chitosan microspheres could release Rg1 and the activity of Rg1 was effective in proliferation and differentiation of hBMSC. Moreover, reintroduction of oxygen to hypoxic cells during reperfusion causes an increase in generation of reactive oxygen species (ROS), which cause damage to proteins, lipids and DNA leading to ischaemia–reperfusion injury, and apoptosis and necrosis [31,32]. After treatment with hypoxia-reoxygenation, the apoptotic rate of hBMSC was significantly increased, but the apoptotic rate of hBMSC was significantly decreased when simultaneous treatment with hypoxia-reoxygenation and Rg1-loaded microspheres stimulation, which implied that Rg1 could decrease the apoptosis in hBMSC induced by hypoxia-reoxygenation. *In vitro* experiments indicated that Rg1-loaded microspheres were safe and effective.

In conclusion, according to the promoting proliferation and differentiation of Rg1-loaded alginate-chitosan microspheres, Rg1-loaded microsperes could be used as a carrier in Rg1 delivery and may play an important role in the treatment of central nervous system diseases. In future, we will conduct a model (*in vivo*) study on Rg1-loaded microspheres.

## Abbreviations

Bax: Diphenhydramine
Bcl-2: B-cell lymphoma-2
bFGF: Basic fibroblast growth factor
BM: bone marrow
BMSC: bone marrow stromal cell
CT: Computed tomography
DMEM: Dulbecco minimum essential medium
GAPDH: Glyceraldehyde-3-phosphate dehydrogenase
GFAP: glial fibrillary acidic protein
hBMSC: human bone marrow stromal cell
NSE: Neuron Specific enolase
PB: Phosphate buffer
RT-PCR: Real time PCR
Rg1: The ginsenoside Rg1
TBST: Tris buffer solution with 0.2% tween
U: Unit
